# Weighting bias and inflation in the time of COVID-19: evidence from Swiss transaction data

**DOI:** 10.1186/s41937-020-00057-7

**Published:** 2020-09-16

**Authors:** Pascal Seiler

**Affiliations:** 1 ETH Zurich, KOF Swiss Economic Institute, Leonhardstrasse 21, Zurich, CH–8092 Switzerland; 2 https://www.pascalseiler.ch

**Keywords:** Inflation, Consumption expenditure, Card transaction data, High-frequency data, COVID-19, C43, E21, E31, E37

## Abstract

Sharp changes in consumer expenditure may bias inflation during the COVID-19 pandemic. Using public data from debit card transactions, I quantify these changes in consumer spending, update CPI basket weights and construct an alternative price index to measure the effect of the COVID-induced weighting bias on the Swiss consumer price index. I find that inflation was higher during the lock-down than suggested by CPI inflation. The annual inflation rate of the COVID price index was −0.4*%* by April 2020, compared to −1.1*%* of the equivalent CPI. Persistent “low-touch” consumer behavior can further lead to inflation being underestimated by more than a quarter of a percentage point until the end of 2020.

## Introduction

The COVID-19 pandemic and the measures enacted to contain it have led to a standstill of public life and a severe downturn of economic activity in many countries, including Switzerland. The measures implemented by the Swiss Federal Council—including lock-downs, mobility restrictions, and social-distancing rules—have greatly affected consumer expenditure patterns. Non-essential retail outlets and many service industries such as restaurants and bars as well as entertainment and leisure facilities were temporarily closed. Public transport services were reduced. Only grocery stores, pharmacies, banks, and post offices were allowed to remain open (Eichenauer and Sturm 2020). During the roughly 2-month1 lock-down period, the consumer spending was thus severely restricted.

Sudden and profound changes like these can introduce significant bias in the consumer price index (CPI) used to measure inflation. The CPI is compiled on the basis of expenditure weights that are kept constant within a given year, reflecting the purpose of the index to measure changes in prices only without accounting for adjustments in consumption patterns. Most national statistical offices update their CPI expenditure weights once a year, often with lagged expenditure data^1^. While this practice is reasonable in normal times, it makes inflation indices much harder to interpret during the COVID-19 pandemic (Tenreyro 2020; Lane 2020), as the underlying weighting scheme is no longer representative of what is being consumed or what can be consumed at all in the lock-down period, thus introducing a *weighting bias*^2^ in inflation.

In this paper, I study the effect that biases induced by such changes in spending patterns have on the measurement of inflation in Switzerland during the COVID-19 crisis. For this purpose, I use high-frequency estimates of spending based on transactional data to update CPI basket weights and compute an alternative price index based on such “COVID weights.”

I find that inflation was higher during the lock-down than suggested by CPI inflation. The annual inflation rate of the COVID price index was −0.4*%* by April 2020, compared to −1.1*%* of the equivalent CPI. This is a consequence of the relative increase in consumption of “food & non-alcoholic beverages,” which are more inflationary than other spending categories.

Moreover, a persistent change in consumer behavior—driven by “low-touch” considerations due to new working habits, prolonged uncertainty, and the lifestyle adopted during the lock-down period—is likely to keep underestimating short- to medium-term inflation. The inflation forecast based on an alternative “low-touch” consumption basket is over a quarter of a percentage point higher than a comparable CPI forecast for the rest of 2020.

This study is closely related to two current research trends. First, it contributes to the rapidly growing literature making use of alternative and high-frequency data sources to track consumer expenditure. In the rapidly evolving COVID-19 crisis, there is a great need for reliable data that is available in almost real-time. Official economic statistics, however, are usually published with a considerable lag. This has led many researchers to explore alternative and high-frequency data sources to track the pandemic and its effects. Transactions’ data from banks and other financial institutions have proved to be particularly fruitful sources and were used for the analysis of consumer spending, among others, by Baker et al. (2020) and Chetty et al. (2020) (for the USA), Chen et al. (2020) (for China), Andersen et al. (2020) (for Denmark), Bounie et al. (2020) (for France), Carvalho et al. (2020) (for Spain), and Hacioglu et al. (2020) (for the UK).

Second and more specifically, it contributes to studies of inflation and potential biases of it during the COVID-19 crisis. My results are consistent with the analytical argument of (Diewert and Fox 2020) who show that a downward bias in consumer price indices may result from current calculation methodologies. Using scanner data of fast-moving consumer goods in the UK, (Jaravel and O’Connell 2020) empirically document a spike in inflation in the first month of lock-down. Using official price indices and updating CPI weights in a similar way to this study, (Cavallo 2020) finds comparable results for the USA but overall mixed international evidence.

In light of the fact that the CPI is an essential tool for economic policy making, my results have important implications for the crisis period and beyond. They provide evidence that conventional price measures have underestimated inflation during the crisis. By unveiling this, I hope that my results contribute to the assessment of inflation in times of economic turmoil. Beyond, they raise conceptual issues concerning adequate price measurement. While most of the changes in prices and consumption expenditure can be expected to reverse once the crisis is over, some of them may be more persistent. Considering current calculation methodologies, this can make official CPI measures less informative, in particular during the transition period. In response to this challenge, the use of high-frequency and alternative data sources on both prices and consumer spending may become key to producing a more robust and informative consumer price index in the future.

The remainder of this paper is organized as follows. In Section 2, I describe how I measure changes in consumer spending, update CPI weights, and construct the alternative COVID price index. Section 3 estimates the effects of the COVID-induced weighting bias on Swiss consumer price index. Section 4 assesses how a lasting “low-touch” consumer behavior will affect inflation in the short to medium term. Section 5 concludes.

## Data and methodology

**Fig. 1:**
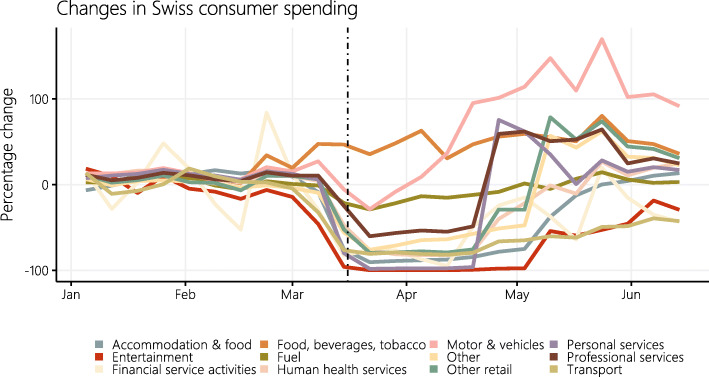
Changes in Swiss consumer spending before and during the COVID-19 crisis as measured by debit card transaction volumes. Cumulative expenditure change across categories of goods and services in Switzerland since January 2020 (normalization month). The vertical dotted line marks the declaration of the *extraordinary situation* by the Swiss Federal Council on March 16, 2020

**Constructing the COVID weights** To construct the COVID basket weights, I use weekly data on Swiss debit card expenditure that are publicly available as part of the Consumption Monitoring for Switzerland^3^. They are produced using transactions of debit cards issued by banks to their customers in Switzerland and include debit card payments at points of sale such as grocery stores or service providers (e.g., hairdressers, restaurants, or petrol stations). Figure 1 depicts the change in Swiss consumption patterns since January 2020 based on these debit card expenditures. The vertical dotted line coincides with March 16, when the *extraordinary situation* was declared by the Swiss Federal Council and when most shops, restaurants, and leisure facilities were temporarily closed.

Three observations stand out^4^. First, there was a massive drop in consumer spending which started even before the introduction of lock-down measures in March 16. This is suggestive of uncertainty and consumer confidence being substantial drivers of the fall in consumption expenditure. Second, different expenditure categories are affected differently by the official measures. While it was virtually impossible to spend money on entertainment or personal services, consumption expenditure for groceries has increased significantly before and during the lock-down period. Third, expenditure categories recover heterogeneously after the lock-down. Expenditure on personal and professional services (including hairdressers) spiked at the end of April, when a first opening step was taken. “Other retail” (including garden stores, clothing, and furniture) is stepwise retracing the two relaxations of April 27 and May 11. While most categories are at least partially recouping their losses, “accommodation & food” and “transport” are recovering only very slowly and are currently still well below their levels at the beginning of the year.

I combine these estimates for consumer spending with official CPI data from the Swiss Federal Statistical Office. In particular, I use the sectoral CPI series that form the lowest level of disaggregation of the CPI (i.e., *expenditure items*, not seasonally adjusted), as well as the latest available weights in the official CPI basket (i.e., expenditure weights for 2020).

Matching debit card transaction categories with the CPI expenditure items requires some assumptions. Table 1 shows the correspondence table for the CPI main groups.

**Table 1:** Matching Swiss CPI main groups and debit card transaction categories

			Weight change
	CPI main group	Debit card transaction category	(Apr vs. Jan 2020, in %)
01	Food & non-alcoholic beverages	Food, beverages, tobacco	36.0
02	Alcoholic beverages & Tobacco	Food, beverages, tobacco	36.0
03	Clothing & footwear	Other retail	−89.7
04	Housing & energy	Other, None	−7.93
05	Furniture & home maintenance	Other, other retail, professional services	−83.9
06	Health	Human health services	−98.2
07	Transport	Fuel, motor & vehicles, transport	−17.8
08	Communications	Other retail	−89.7
09	Recreation & culture	Entertainment, other, other retails,	−92.6
		professional services, transport	
10	Education	None	0.0
11	Restaurants & hotels	Accommodation & food	−98.2
12	Miscellaneous goods & services	Financial service activities, other, other retails,	−83.3
		personal services, professional services	

About five categories are closely matched in both datasets. In particular, there are almost one-to-one mappings for the main groups “health,” “transport,” and “restaurants & hotels.” For both “food & non-alcoholic beverages” and “alcoholic beverages & tobacco,” I use the debit card transaction category “food, beverages, tobacco.” Further, “clothing & footwear,” “furniture & home maintenance,” and “communications” are contained in “other retail.” Items of “recreation & culture” and “miscellaneous goods & services” are matched with multiple transaction categories. Finally, as I do not find any corresponding debit card transaction categories for rents in “housing & energy” and all items in “education,” I assume that expenditure for these items remains unchanged.

To estimate the expenditure shares in the COVID basket, I start with the latest official CPI expenditure weights *w*_*i*,0_, multiply them by the average percentage change in the corresponding expenditure category each month and normalize them as a share of the total. Formally, the COVID weight of CPI expenditure item *i* in month *t* is thus given by

1 $$ w'_{i,t} = \frac{w_{i,0} \Delta e_{i,t}}{{\sum\nolimits}_{i} w_{i,0} \Delta e_{i,t}},  $$

where $\Delta e_{i,t} = \frac {P_{i,t} Q_{i,t}}{P_{i,0} Q_{i,0}}$ is the change in consumer expenditure since January 2020 (base month) as measured by debit card purchases volumes *P*_*i*,*t*_*Q*_*i*,*t*_. Equation 1 highlights the fact that these are *relative* weights, so the importance of a category in the basket can change even when its expenditure does not.

**Constructing the COVID price index** Two concepts of price index calculation are most common: the Laspeyres index and the Paasche index. Conceptually, they differ in one important aspect. The Laspeyres index answers the question of how much the old basket of goods and services costs at current prices. The Paasche index, on the other hand, answers the question of how much the current basket at current prices costs in relation to the current basket at old prices.

Given the COVID-induced monthly changes in consumption expenditure, I favor the use of a Paasche index for the purpose of this study. Comparing index values with the same underlying weighting reduces fluctuations due to changes in expenditure quantities and allows to capture more precisely changes in the price level only. This approach herein differs from similar contributions of the literature, in particular from (Cavallo 2020) who, through the monthly variation of both prices and weights, blurs the boundary between the CPI as a tool for pure price measurement and the CPI as a mere turnover statistic.

Consequentially, I compute the COVID price index as the weighted sum of sectoral CPI indices using two distinct weighting schemes in any month, namely

2 $$ I_{t, w = t} = \sum\limits_{i=1}^{12} w'_{i,t} I_{i,t}  $$

and

3 $$ I_{t-k, w = t} = \sum\limits_{i=1}^{12} w'_{i,t} I_{i,t-k}  $$

where *k*∈{1,12} for the calculation of either the monthly or annual inflation rates. These inflation rates are then calculated by comparing the indices of the same weighting scheme, i.e.,

4 $$ \pi_{t, t-1} = \frac{I_{t, w = t} - I_{t-1, w = t}}{I_{t-1, w = t}} \cdot 100  $$

in case of monthly inflation, and

5 $$ \pi_{t, t-12} = \frac{I_{t, w = t} - I_{t-12, w = t}}{I_{t-12, w = t}} \cdot 100  $$

in case of annual inflation.

## Inflation with COVID consumption

**Fig. 2:**
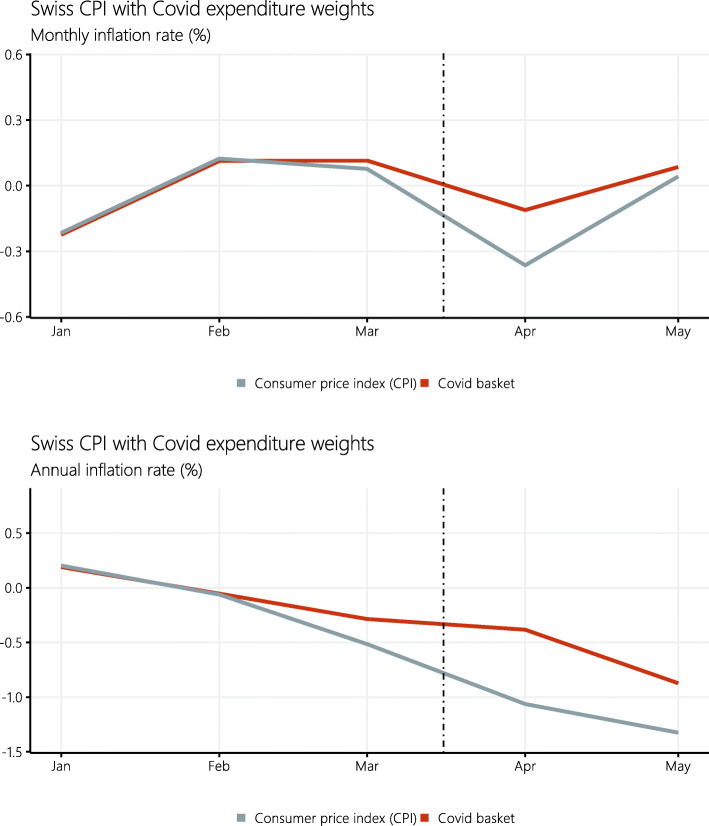
Swiss consumer price index (CPI, not seasonally adjusted) and the COVID index construed using estimates of the consumption expenditure shares during the COVID-19 crisis (“COVID basket”). The vertical dotted line marks the declaration of the *extraordinary situation* by the Swiss Federal Council on March 16, 2020

Figure 2 and Table 2 show the alternative COVID price index and thus illustrate the effect of the COVID-induced changes in consumer spending on both the monthly and annual inflation rates of the Swiss consumer price index.

**Table 2:** Swiss inflation rates during the COVID-19 crisis

	Monthly inflation		Annual inflation	
	CPI	COVID basket	CPI	COVID basket
January	–0.22	–0.22	0.19	0.19
February	0.13	0.11	–0.07	–0.05
March	0.08	0.11	–0.51	–0.29
April	–0.36	–0.11	–1.05	–0.38
May	0.05	0.09	–1.32	–0.87

Monthly and annual inflation rate in the not seasonally adjusted Swiss CPI and the COVID index construed using estimates of the consumption expenditure shares during the COVID-19 crisis

The Swiss CPI was low but relatively stable in the beginning of the year, before it started showing deflation from February onward. During the COVID-19 crisis, it contracted strongly. It fell by −0.36*%* in April 2020 compared with the previous month. Inflation was −1.05*%* compared with the same month of the previous year. After lock-down in May 2020, it increased slightly by 0.05%.

The COVID price index is similar to the CPI in the months before the lock-down. The average difference between the two indices amounts to 0.07 percentage points, which is consistent with (Diewert and Fox 2020) who estimate a substitution bias of 0.13*%* for the Swiss CPI in the period 1993–2002. By contrast, the COVID price index was considerably and consistently higher during the lock-down. It contracted by −0.11*%* only in April 2020 compared with the previous month, and inflation was −0.38*%* compared with the same month of the previous year. After lock-down in May 2020, it increased slightly by 0.09*%*.

To illustrate what is driving these results, Table 3 shows the category weights and incidence details for April 2020. The second column has the monthly CPI sector inflation used in both the official and COVID price index. The third and fourth columns show the weights of the CPI and COVID basket in each category. Finally, the last two columns show the incidence that each category has on the total monthly inflation rate. The incidence is the monthly inflation rate multiplied by the weight. Therefore, the sum of all the category incidence numbers is equal to the monthly inflation rate.

**Table 3:** Swiss CPI weights and incidence in April 2020

	Monthly	Weight		Incidence	
Main group	CPI inflation	CPI	Covid basket	CPI	Covid basket
Food and non-alcoholic beverages	0.71	10.54	23.70	0.07	0.17
Alcoholic beverages and tobacco	–0.47	2.76	6.20	–0.01	–0.03
Clothing and footwear	0.26	3.40	1.52	0.01	0.00
Housing and energy	–0.19	24.96	40.27	–0.05	–0.08
Household goods and services	–0.80	3.79	1.69	–0.03	–0.01
Healthcare	–0.08	15.69	6.11	–0.01	–0.01
Transport	–1.74	10.97	7.90	–0.19	–0.14
Communications	–0.06	2.94	4.75	–0.00	–0.00
Recreation and culture	–0.89	8.37	0.08	–0.07	–0.00
Education	0.00	1.00	1.61	0.00	0.00
Restaurants and hotels	–0.75	9.46	2.04	–0.07	–0.02
Other goods and services	–0.09	6.12	4.13	–0.01	–0.00

The CPI weight is the share of expenditure in a given category over total expenditures. The incidence is the monthly inflation rate multiplied by the weight. The sum of all the category incidence numbers is equal to the monthly inflation rate

Note that main groups whose spending does not change over time per assumption (e.g., “education”) can have different COVID weights due to the normalization of the COVID basket as a share of total basket expenditure. Table 3 illustrates that the result is driven by shifts in relative basket weights. The COVID inflation rate is higher than CPI inflation because the index based on COVID weights gives more weight to main groups that have a positive inflation rate, and less weight to categories experiencing deflation. In particular, the weight for “food & non-alcoholic beverages” rose from 10.54 to 23.70%, increasing the incidence of this category from 0.07 to 0.17. At the same time, the weight for the deflationary category “recreation & culture” fell from 8.37 to 0.08%, virtually eliminating the influence of this main group on the total monthly inflation rate.

Altogether, the weighting bias in official CPI statistics seems to have underestimated inflation. After adjusting for the change in consumer spending during the pandemic, the inflation rate of the COVID price index is two thirds of the percentage points higher and lies at −0.38*%* in April 2020. This result is driven by the relative weight shifts, positively reinforcing inflationary CPI main groups, and negatively reinforcing deflationary main groups.

## Inflation with low-touch consumption

Having quantified the effect during the lock-down period raises the question of how any lasting change in consumer behavior will affect inflation in the short to medium term.

Most of the changes in consumer spending during the COVID-19 crisis are obviously driven by the containment measures and forced closures of many retail sectors. Once these restrictions are lifted, catch-up effects can be expected, and consumer spending will gradually converge back to pre-crisis levels, as apparent in Fig. 1.

**Fig. 3:**
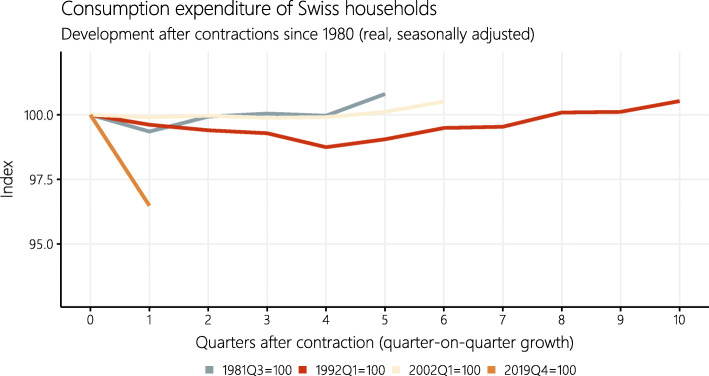
Development of Swiss households’ final consumption expenditure (real, seasonally adjusted) after contractions (quarter-on-quarter growth) since 1980. Included are contractions with negative quarterly growth rates in at least two consecutive quarters

Nevertheless, an immediate rebound and normalization to pre-crisis levels seems unlikely. Rather, getting back to pre-COVID consumption patterns is likely to be a long and difficult task. Figure 3 compares the decline in consumer spending in the COVID-19 crisis with the earlier contractions of Swiss households’ final consumption expenditure since 1980.

So far, there have been a total of three downturns (of two consecutive quarters or more) in consumption, related to the second oil crisis (1981), the Swiss real-estate crisis (1992) and the dot-com bubble (2002). Even if consumers return to normality at different speeds depending on the type of disruption that hit the economy, Fig. 3 illustrates how unprecedented the current downturn in consumption is. Measured against earlier recoveries, the normalization after the current crisis is likely to extend over several quarters.

Moreover, consumer behavior may have changed persistently during the crisis, and henceforth be driven by “low-touch” considerations. This kind of “low-touch” consumption is characterized first and foremost by continuing uncertainty about the spread of the virus, changed working habits and the lock-down lifestyle^5^.

The lock-down has led to working from home in many professions. Once efficient conditions for teleworking are in place, they are likely to stay, which in turn creates more opportunities for eating at home, and reduces eating out. This negatively affects the hospitality and tourism sectors, which have already been severely impacted by the far-reaching travel freeze. In view of the great uncertainties regarding the spread of the virus and the travel preparations which have become fairly complex (administrative effort to obtain information on the local situation and regulations), it seems plausible that expenditure for travel and tourism will remain subdued in the short and medium term. Further, the lock-down period has had a negative impact upon leisure activities. Digital activities such as online gatherings, at-home entertainment as well as remote learning and exercise have emerged. Outdoor and fitness activities have replaced going to the gym. These activities are likely to remain strong and reduce the share of spending on traditional “recreation & culture” activities reflected in the CPI.

In the following, I will examine the effects this kind of prolonged change in consumer spending has on inflation in the short to medium term. I do so by producing inflation forecasts based on disaggregated CPI data, which I aggregate using two different weighting schemes: the official CPI weights, and the expenditure weights implied by the “low-touch” scenario.

Table 4 compares the latest CPI weights with the weights implied by this “low-touch” scenario. I calculate the latter by applying fixed markups (or markdowns) to the official CPI weights for 2020.

**Table 4:** Swiss CPI weights and “low-touch” weights

Main groups	CPI weights	Low-touch weights	Low-touch add-on
Food and non-alcoholic beverages	10.31	12.00	15%
Alcoholic beverages and tobacco	2.91	2.94	
Clothing and footwear	3.59	3.63	
Housing and energy	25.31	25.60	
Household goods and services	3.67	3.71	
Healthcare	14.33	14.49	
Transport	11.58	11.04	–7.7%
Communications	3.11	3.14	
Recreation and culture	8.43	7.45	–3.8%
Education	1.05	1.06	
Restaurants and hotels	9.45	8.61	–10%
Other goods and services	6.25	6.32	

The CPI weight is the share of expenditure in a given category over total expenditures. The low-touch weights are the assumed expenditure shares in the “low-touch” scenario. They are calculated by adding fixed markups (or markdowns) to the CPI weights for 2020. the fourth column gives the average add-on per main group. Weights are normalized and applied from June 2020 until the end of the forecast period

I assume that spending on groceries will continue to fall and approach the original level, but then remain at +15*%* compared to January 2020. Conversely, out-of-home expenditure in restaurants and hotels will fall by 10%. In addition, consumption expenditure on selected transport services (air transport and package holidays in particular) and leisure activities (such as leisure courses, cinema, theater, and concert) are assumed to decrease, leading to the overall differences of −7.7*%* and −3.8*%* in the corresponding main groups.

As the imposed change in weight is negative overall and the weights are normalized as a share of total expenditure, the relative “low-touch” weights of the otherwise unchanged main groups end up marginally increased. These weighting schemes are applied from June 2020 until the end of the forecast period.

The disaggregated forecasts of the CPI items are based on univariate ARIMA models (similar to Huwiler and Kaufmann (2013)).

For each expenditure item, the model is selected in two steps. First, the statistical properties of each price series in analyzed. For simplicity, I assume that all items are integrated of order one and use them accordingly in first log-differences. Further, seasonal patterns are detected through inspecting the autocorrelation function (ACF) and considering the price collection frequency. Second, the lag selection of each model is automated using the Schwarz information criterion.

I forecast the CPI items with the monthly series from May 2000 (where available) to December 2021 and then aggregate the forecasts using the weighting schemes based on the two scenarios. For the benchmark scenario, I use the official CPI expenditure weights for 2020 throughout. For the “low-touch” scenario, I take the COVID weights from Section 3 until May 2020 and apply the low-touch weights (Table 4) from June 2020 onwards.

**Fig. 4:**
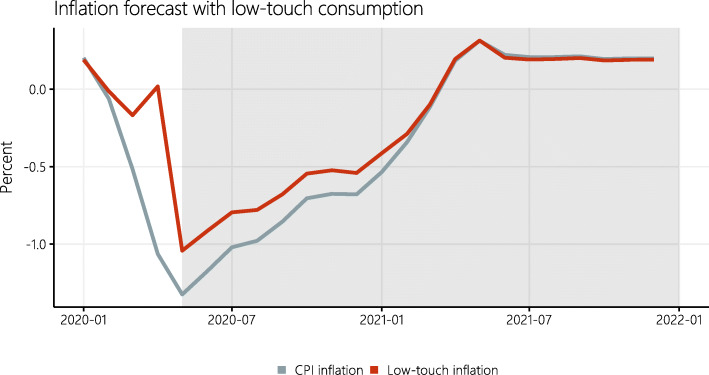
Comparison of inflation forecasts with official CPI weights and with “low-touch” consumption

Both indices are shown in Fig. 4 as Laspeyres indices. I use Laspeyres indices for this counterfactual analysis in order to ease and ensure direct comparability between my results and other forecasts. The Swiss CPI is itself a Laspeyres index, which is why forecasts by professional forecasters, and policymakers are usually based on this calculation method, too.

CPI inflation is projected to increase gradually from its low in May 2020 (−1.3*%*) but will remain considerably below 0% well into 2021. An average inflation rate of −0.72*%* is expected for 2020, and 0.12*%* for 2021. In contrast, inflation under the “low-touch” scenario is persistently higher. Particularly in the current year, inflation rates differ significantly. In the low-touch scenario, the average annual inflation is −0.43*%*. Only in the course of next year, the two forecasts converge.

Thus, without taking into account a sustained change in consumer behavior based on low-touch considerations, the official CPI statistics risks underestimating inflation in the current year by more than a quarter of a percentage point.

It is difficult to say to what extent the presumed changes in consumer behavior are really permanent. It is probable that the longer the crisis lasts, the more the new behaviors will gradually become the new normality and continue after the pandemic. The extent to which consumer behavior and spending will change in the medium to long term after the recession is therefore likely to depend to a large extent on the further course of the pandemic, as well as on how the new work experiences are integrated into existing work habits and consumers’ assessment of their future prospects.

## Conclusion

Measuring and interpreting inflation is challenging during economic disruptions in general and the COVID-19 crisis in particular. Consumer spending is greatly affected by the pandemic containment measures, introducing a weighting bias into the measurement of CPI inflation.

Using public data from debit card transactions in Switzerland, I estimate the changes in consumption expenditure during the COVID-19 crisis and construct an alternative price index with updated COVID consumption weights to study the effect of these sources of bias on Swiss CPI inflation.

I find that COVID inflation was higher than suggested by CPI inflation. By April 2020, the annual inflation rate of the COVID price index was −0.38*%*, compared to −1.05*%* of the CPI. This is a consequence of the relative increase in consumption of “food & non-alcoholic beverages.”

Moreover, a persistent change in consumer behavior—driven by “low-touch” considerations due to new working habits, prolonged uncertainty, and the lifestyle adopted during the lock-down period—keeps underestimating short- to medium-term inflation throughout the year by more than a quarter of a percentage point. In 2020, CPI inflation is projected to average −0.72*%*, while COVID inflation is projected to average −0.43*%*.

**Fig. 5:**
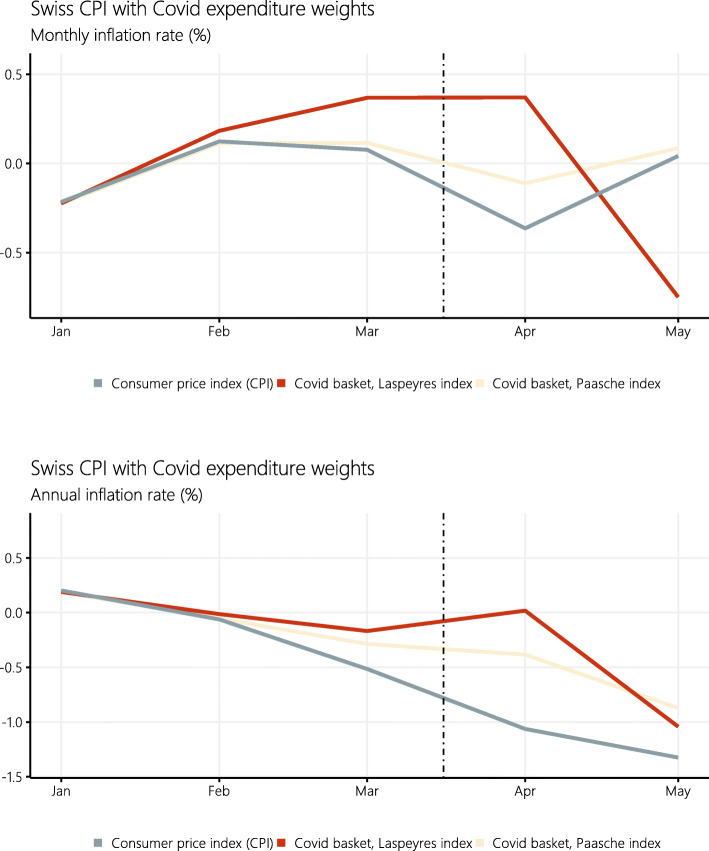
Swiss consumer price index (CPI, not seasonally adjusted) and COVID price indices, calculated once as Paasche index and once as Laspeyres index. The vertical dotted line marks the declaration of the *extraordinary situation* by the Swiss Federal Council on March 16, 2020

**Table 5:** Timeline of the events and measures taken by the Swiss Federal government. Compiled from the ordinances and media releases of the Swiss Federal Council, see https://www.admin.ch/gov/en/start/ documentation.html. Situation as of June 24, 2020

28 February	The Swiss Federal Council categorizes the situation in Switzerland as *special* in terms of the Epidemics Act. Events with more than
	1000 persons are prohibited effective immediately. Among other things, the Basel Carnival and the Geneva Motor Show are canceled.
13 March	The government announces the closure of the schools on Monday, March 16. Events with more than 100 people are prohibited.
	Restaurants, bars, and discos are limited to 50 people.
16 March	The Federal President declares the *extraordinary situation*, allowing the Federal Council to order the introduction of uniform measures
	in all cantons. All public and private events are prohibited. Shops, restaurants, and leisure facilities must close. The lock-down also
	applies to schools and businesses at which the recommended distance cannot be maintained (e.g., hairdressers and cosmetic
	studios). Only grocery stores and health facilities remain open. Border controls at the borders with Germany, Austria, and France
	were introduced, and entry bans imposed, albeit with exceptions. Border checks at the Italian border were already introduced
	at an earlier stage. Up to 8000 members of the armed forces were deployed to assist the cantons at hospitals and with logistics and security.
27 April	First step towards opening: hairdressers, DIY stores, and garden centers may resume operations with protection concepts.
11 May	Shops, restaurants, public markets, and museums may reopen. Primary and secondary schools can again teach on site.
6 June	Events with up to 300 people are permitted again. Mountain railways, camping sites, zoos, and leisure facilities may open.
	Secondary, vocational, and higher education establishments may resume teaching.
15 June	The borders to all states within the EU/EFTA area will be opened completely. Among other things, shopping tourism to Germany
	or Austria is permitted again.
19 June	Return from the extraordinary to the special situation. The cantons will have a greater say and more room for maneuver. In public
	spaces, the minimum distance is reduced from 2 to 1.5 m. Restaurants will be allowed to move their tables closer together, while
	at the same time the Swiss midnight curfew will be lifted. Meetings and events for up to 1000 people are again permitted. Masks are
	compulsory at rallies. The recommendation to work from home if possible is repealed.

In light of the fact that the CPI is an essential tool for economic policy making, my results have important implications for the crisis period and beyond. They provide evidence that conventional price measures have underestimated inflation during the crisis. By unveiling this, I hope that my results contribute to the assessment of inflation in times of economic turmoil. Beyond, they raise conceptual issues concerning adequate price measurement. While most of the changes in prices and consumption expenditure can be expected to reverse once the crisis is over, some of them may be more persistent. Considering current calculation methodologies, this can make official CPI measures less informative, in particular during the transition period. In response to this challenge, the use of high-frequency and alternative data sources on both prices and consumer spending may become key to producing a more robust and informative consumer price index in the future.

## Appendix

### A The stages of Swiss lock-down

### B Comparison of Paasche and Laspeyres price indices

## Data Availability

The data analyzed during the current study are available in my GitHub respository, https://github.com/pascalseiler/CovidCPIBias.

## Abbreviations

ACF: Autocorrelation function
ARIMA: Autoregressive-integrated moving average
CEPR: Centre for Economic Policy Research
COVID-19: Coronavirus disease 2019
CPI: Consumer price index
DIY: Do it yourself
EFTA: European Free Trade Union
ETH: Eidgenössische Technische Hochschule
EU: European Union
FSO: Federal Statistical Office
KOF: Konjunkturforschungsstelle
NBER: National Bureau of Economic Research
UK: United Kingdom
USA: United States of America

## Footnotes

1 The Swiss Federal Statistical Office (FSO) updated the CPI weights for 2020 in December 2019 using expenditure information collected back in 2017 and 2018. The main source for calculating the basket weights is the Household Budget Survey (HBS), conducted annually by the FSO among private households with permanent residence in Switzerland.

2 Bryan and Cecchetti (1993) conceptually distinguish between two sources of bias in price indices: biases resulting from the expenditure-based weighting scheme (*weighting bias*) and from errors in measuring prices (*measurement bias*). Apart from the weighting bias, which is the focus of this paper, the COVID-19 crisis may also have led to a measurement bias in the CPI. Indeed, the measures enacted by the Federal Council have impeded price collection. In April 2020, about 20% of the prices usually surveyed in that month could not be observed (FSO 2020). Instead, prices were collected remotely and imputation techniques were applied. Using online data for prices, (Alvarez and Lein 2020) show that prices declined by more than what is suggested by the prices underlying the CPI during the lock-down in Switzerland.

3 The Consumption Monitoring for Switzerland is a project powered the University of St. Gallen (Prof. Martin Brown, Prof. Matthias Fengler) and Novalytica together with Dr. Robert Rohrkemper (Distinguished Expert, Senior Data Scientist at Worldline) and Prof. Rafael Lalive (University of Lausanne). See http://monitoringconsumption.org/switzerland.

4 These findings are corroborated by international studies tracking consumption similarly with high-frequency credit and debit card transactions. See Baker et al. (2020) and (Chetty et al. 2020) for the USA, (Chen et al. 2020) for China, (Andersen et al. 2020) for Denmark, (Bounie et al. 2020) for France, (Carvalho et al. 2020) for Spain, and (Hacioglu et al. 2020) for the UK. Beyond, (Coibion et al. 2020) find comparable survey-based estimates.

5 For survey evidence in support of this “low-touch” consumption scenario, see for example (Boston Consulting Group 2020) or (McKinsey & Company 2020).
